# Draft Genome Sequence of Yersinia ruckeri Strain FMV-22, Isolated from Diseased Rainbow Trout (Oncorhynchus mykiss) in Peru

**DOI:** 10.1128/MRA.01389-19

**Published:** 2020-01-16

**Authors:** Fernando Mesías, Diego Gerpe, Luis Llanco, E. Serrano-Martínez, Jesús L. Romalde

**Affiliations:** a Facultad de Medicina Veterinaria y Zootecnia, Universidad Peruana Cayetano Heredia, Lima, Peru; b Departamento de Microbiología y Parasitología, CIBUS-Facultad de Biología, Universidade de Santiago de Compostela, Santiago de Compostela, Spain; University of Arizona

## Abstract

Yersinia ruckeri is a significant and frequent bacterial fish pathogen in Peru. We report the draft genome sequence of strain FMV-22, isolated from a diseased rainbow trout, which consists of 3.84 Mb, with a G+C content of 47.45% and 3,765 protein-coding genes.

## ANNOUNCEMENT

Yersinia ruckeri is a Gram-negative enterobacterium that causes enteric redmouth disease (ERM) in a wide variety of fish, especially affecting salmonids like rainbow trout (Oncorhynchus mykiss) (1). Fish infected with this bacterium present hemorrhages in the mouth and eyes, lethargy, exophthalmia, and petechial hemorrhages in different internal organs (2, 3). These signs are related to virulence factors like hemolysins, siderophores, and invasins, among others (3–5). In many parts of the world, prevention and control of Y. ruckeri usually involve the application of vaccines or antibiotics; however, strains resistant to different antimicrobials have appeared in some countries (6–9). In Peru, this bacterium is one of the main pathogens that harms the production of rainbow trout, and it is treated mainly through the use of antibiotics, because no vaccines are currently available in this country. Yersinia ruckeri strain FMV-22 was isolated from diseased rainbow trout (O. mykiss) raised on a fish farm located in Canta-Lima, which presented frequent ERM outbreaks despite receiving antibiotic treatments. The isolate was obtained from a kidney sample inoculated onto Trypticase soy agar (Liofilchem, Italy), after 24 h of incubation at 25°C. The strain was stored at −80°C in Trypticase soy broth supplemented with 20% glycerol.

For DNA extraction, a single colony of Y. ruckeri FMV-22 was inoculated onto Trypticase soy agar and incubated at 25°C for 24 h. The bacterial culture was resuspended in sterile saline solution (0.85% [wt/vol] NaCl), and genomic DNA was extracted using the DNeasy blood and tissue kit (Qiagen, Germany) following the manufacturer’s instructions. The DNA was sent to Fisabio (Valencia, Spain) for whole-genome sequencing. A genomic DNA library was prepared using the Nextera XT library preparation kit (Illumina, San Diego, CA, USA) following the manufacturer’s protocol. The library was then subjected to paired-end sequencing (2 × 300 bp) using an Illumina MiSeq sequencer with 100× coverage, producing 1,923,132 paired-end reads (∼242-bp read length). Reads were *de novo* assembled into 106 contigs (>200 bp) using SPAdes 3.12 (10), and the quality of the assembly was evaluated with QUAST 5.0.2 (11). The resulting draft genome consists of 3,842,153 bp, with an average G+C content of 47.45%. The contig *N*_50_ value is 152,665 bp, with the largest contig being 433,565 bp. Annotation of the genome was performed using the Rapid Annotations using Subsystems Technology (RAST) 2.0 server (12), and tRNAs were analyzed with tRNAscan-SE 2.0 (13). Default parameters were used for all software. The genome of FMV-22 contains 3,765 coding sequences and 73 tRNAs.

The annotation revealed that the Y. ruckeri strain FMV-22 genome contains a total of 24 coding sequences associated with resistance to antibiotics and toxic compounds, including copper homeostasis, cobalt-zinc-cadmium resistance, fluoroquinolone resistance, copper tolerance, adaptation to d-cysteine, and a beta-lactamase. Moreover, 4 genes related to adhesion and 13 coding sequences linked to invasion and intracellular resistance were identified. In addition, RAST detected 19 coding sequences associated with iron acquisition and metabolism, including 9 genes linked to siderophores.

This genome sequence information contributes to comparative genomic analysis and to the study of virulence and antimicrobial resistance factors of this important fish pathogen.

### Data availability.

The genome sequence of Yersinia ruckeri strain FMV-22 has been deposited in DDBJ/EMBL/GenBank under accession number VDHI00000000. The version described in this paper is VDHI01000000. The raw reads have been submitted and are available under Bioproject number PRJNA546113 and Biosample number SAMN11952176.
